# Functional capacity in Peruvian people with Alzheimer's disease and frontotemporal dementia

**DOI:** 10.1002/alz.70979

**Published:** 2025-12-16

**Authors:** Nilton Custodio, Gregory Brown, Belén Custodio, Rosa Montesinos, Milagros Nuñez‐Huanca, Pamela Bartolo, Zadith Yauri, Katherine Agüero, Graciet Verastegui, Agustin Ibañez

**Affiliations:** ^1^ Instituto Peruano de Neurociencias Unidad de diagnóstico de deterioro cognitivo y prevención de demencia Lince Peru; ^2^ Department of Neurology University of California San Francisco California USA; ^3^ Department of Internal Medicine Medstar Washington Hospital Center Washington District of Columbia USA; ^4^ Centro de Investigación del Envejecimiento, Facultad de Medicina Universidad de San Martín de Porres La Molina Peru; ^5^ Equilibria Unidad de Investigación y Docencia Santiago de Surco Peru; ^6^ Latin American Brain Health Institute (BrainLat) Universidad Adolfo Ibañez Santiago de Chile Chile; ^7^ Cognitive Neuroscience Center Universidad de San Andres Buenos Aires Argentina; ^8^ Global Brain Health Institute (GBHI) Trinity College Dublin Dublin Ireland

**Keywords:** Alzheimer's disease, dementia subtypes, frontotemporal dementia, functional capacity, instrumental activities of daily living, Latin America, neuropsychological assessment, Peru

## Abstract

**INTRODUCTION:**

Functional impairment across degenerative dementias remains understudied in Latin American contexts. We aimed to assess total and item‐level functional performance, determine specific instrumental activities, and identify differences in functional profiles between groups.

**METHODS:**

In this cross‐sectional study, 1445 participants were classified according to the Pfeffer Functional Activities Questionnaire (PFAQ). Statistical comparisons were adjusted for age, sex, and education using linear regression residuals and corrected for multiple comparisons.

**RESULTS:**

Functional impairment increased progressively from normal cognition to dementia. Alzheimer's disease (AD) and frontotemporal dementia (FTD) groups showed significantly greater total PFAQ scores compared to controls and mild cognitive impairment (MCI). Distinct profiles of impairment emerged: AD was more associated with memory‐dependent tasks, while FTD showed disproportionate deficits in executive and social activities.

**DISCUSSION:**

Functional abilities are differentially impacted in AD and FTD within the Peruvian population. Our findings highlight the importance of item‐level functional evaluation to support early detection and subtype differentiation of dementia in underserved regions.

**HIGHLIGHTS:**

- We characterized and compared functional abilities among Peruvian older adults with normal cognition and neurodegenerative disease in different stages.
- PFAQ scores were analyzed across dementia stages in a Peruvian population.
- Functional abilities were differentially impacted in Peruvian participants with AD compared to FTD.
- AD was more associated with memory‐dependent tasks, while FTD showed disproportional deficits in executive and social activities.

## 1 INTRODUCTION

Dementia is one of the most significant public health challenges globally, and Latin America is experiencing a sharp increase in its prevalence due to rapid population aging.^1^, ^2^ In Peru, this demographic transition is compounded by socioeconomic and healthcare disparities, potentially shaping unique manifestations of dementia in this setting.^3^ Among the core components of healthy aging and dementia is the preservation of functional abilities, which encompass instrumental activities of daily living essential for independence and quality of life.^4^, ^5^ However, the Peruvian population experiences specific cultural, educational, and environmental contexts that may influence how functional abilities decline in aging and across the dementia spectrum. It remains unknown how functional abilities manifest along the continuum from normal aging to neurocognitive disorders, including mild cognitive impairment (MCI), Alzheimer's disease (AD), and frontotemporal dementia (FTD), within Peruvian populations.

Research on functional abilities in aging and dementia in Peru has been scarce^6^ and fragmented to AD.^7^ Existing studies are primarily focused on cognitive screening^8^, ^9^ or prevalence estimates,^10^, ^11^ with limited attention paid to functional profiles across different dementia subtypes. Moreover, most research has not systematically examined how individual instrumental activities are affected differently in healthy aging, MCI, AD, and FTD. There is also a lack of detailed analysis identifying specific activities that may serve as early markers of disease or differentiate between dementia subtypes. Additionally, prior work seldom adjusted analyses for critical sociodemographic variables such as age, gender, and education, factors known to influence both cognitive and functional outcomes in diverse populations. These gaps leave an incomplete understanding of the functional implications of neurodegenerative diseases in Peru, hindering early detection and tailored interventions.

Here we aim to bridge these gaps by characterizing functional abilities among Peruvian older adults across the spectrum from normal cognition to MCI, AD, and FTD. Specifically, our objectives were to (1) compare overall functional performance between healthy controls, individuals with MCI, and patients with AD and FTD; (2) identify which specific instrumental activities are disproportionately impaired relative to total functional scores at different stages of disease; and (3) to determine whether patterns of functional deficits differ between AD and FTD. We also (4) reran all analyses controlling for age, gender, and education to account for potential confounding effects. We hypothesize that patients with dementia will exhibit significant functional impairments compared to controls and MCI and that FTD and AD will show distinct functional profiles, reflecting differences in underlying cognitive and behavioral changes associated with each disorder.

## 2 METHODS

### 2.1 Study design and participants

We conducted an observational, cross‐sectional study with participants at the research unit of the Instituto Peruano de Neurociencias (IPN). Participants were enrolled consecutively between May 2020 and December 2024. Eligibility criteria included (1) an established classification as a control participant or diagnosis of neurocognitive disorder^12^ (MCI^13^ or dementia due to AD^14^ or FTD^15^) based on established clinical criteria, (2) presence of a formal or informal informant/caregiver (see below), (3) over 50 years of age, (4) caregiver ability to complete the questionnaire, (5) speaking Spanish as a primary language, and (6) having a minimum of 4 years of formal education. Caregivers were family members or close friends who had direct contact with the patient for more than 6 h each day for 3 consecutive months prior to the interview. Caregivers were also screened with the Zarit scale^16^ and Patient Health Questionnaire 9^17^ to exclude individuals with caregiver burnout (Zarit > 30) or depression (score > 4). Interviews occurred without the participant present to promote honesty. Patients whose cognitive function could be impaired by certain drugs or by a particular medical condition, including a history of addiction and substance abuse, depression, hypothyroidism, vitamin B12 deficiency, chronic kidney or liver disease, HIV or syphilis neuro‐infections, severe head trauma, and subdural hematoma, were excluded. Additionally, those with a condition that could affect their performance in the cognitive evaluation were excluded (hearing and visual impairments, motor sequelae of cerebrovascular disorder, or traumatic sequelae). Participants were classified based on Clinical Dementia Rating (CDR; mild = 1, moderate/severe = 2 to 3) and clinical diagnosis into six diagnostic groups: controls, MCI, mild AD, mild FTD, moderate/severe AD, and moderate/severe FTD.

**RESEARCH IN CONTEXT**

1. **Systematic review**: Peruvian culture presents specific contexts that may influence how functional abilities decline in aging and across the dementia spectrum; however, it remains unknown how functional abilities manifest along the continuum from normal aging to neurocognitive disorders in Peru.
2. **Interpretation**: We present the first Peruvian cohort with detailed functional capacity across the continuum from normal aging to MCI, AD, and FTD distinguishing the functional profiles of these two neurodegenerative disorders.
3. **Future directions**: Future studies should determine clinically useful thresholds for MCI and dementia, as well as the generalizability of these profiles to other Spanish‐speaking countries in Latin America. Additional culturally relevant variables, such as literacy, rural versus urban residence, and quality of education, should also be considered.

### 2.2 Diagnostic process and final classification

The evaluations were carried out at the IPN research department using a standardized cognitive evaluation process (see clinical assessments) for people with dementia, where each subject underwent clinical and neuropsychological evaluation. The control group consisted of individuals who came to the clinic for cognitive evaluations and had negative results on brief cognitive and functional tests and underwent the same neurocognitive and functional evaluation as the case groups. Often these individuals had subjective cognitive complaints, but some individuals simply wanted a cognitive evaluation. During these same sessions, the informants were interviewed to complete the Pfeffer Functional Activities Questionnaire (PFAQ).

Participants were referred from the IPN neurology service, where all patients underwent a cognitive evaluation during their medical consultation, following a three‐phase process: (1) screening – to detect cognitive impairment, (2) disease classification – diagnostic testing to rule out other causes of dementia, and (3) final classification – dementia subtype and severity of the disease. In the screening phase, the brief cognitive and functional tests used were Rowland Universal Dementia Assessment Scale (RUDAS)^18^ and PFAQ.^19^

After a consensus meeting between neurologists, geriatricians, psychiatrists, and neuropsychologists, the probable type of dementia was defined by applying the diagnostic criteria of Diagnostic and Statistical Manual of Mental Disorders, 5th Edition,^12^ MCI,^13^ AD,^14^ and FTD.^15^ Additionally, the CDR scale^20^ was used for staging of dementia severity: CDR = 0 (control), CDR = 1 (mild dementia), CDR = 2 (moderate dementia), and CDR = 3 (severe dementia). CDR was administered to both participants and informants.

### 2.3 Clinical assessments

For our neurocognitive assessments, we used standard assessments, which have been validated in Peruvian contexts. We used the Peruvian adaptation of the Mini‐Mental State Examination (MMSE),^21^, ^22^ the RUDAS,^23^, ^24^ and the Spanish version 7.0 of the Montreal Cognitive Assessment (MoCA).^25^ Notably, MCI participants only completed MMSE, whereas all other groups completed all three assessments.

For our functional assessment, we used the PFAQ. PFAQ is composed of 11 items assessing Instrumental Activities of Daily Living (I‐ADL). Each item is rated on a four‐point scale from 0 (normal or never did but could do now) to 1 (has difficulty but does by self or never did but would have difficulty now), 2 (requires assistance) and 3 (dependent). Total scores range from 0 to 33, with higher scores indicating higher functional impairment.^19^

### 2.4 Statistical analysis

All analyses were performed in MATLAB R2024a. We evaluated group differences in functional abilities using PFAQ total score and each of the 11 individual items. Participants were classified into six diagnostic groups based on CDR and clinical diagnosis: cognitively normal (control), MCI, mild AD (CDR = 1), mild FTD (FTD Mild, CDR = 1), moderate/severe AD (AD Mod/Severe, CDR ≥ 2), and moderate/severe FTD (FTD Mod/Severe, CDR ≥ 2). Outcome variables (both total scores and individual items) were adjusted for age, sex, and education using linear regression. We applied this adjustment prior to group analyses to leverage the full dataset for covariate correction, which also simplifies interpretation and plotting. All plots show the marginal mean (outcome measures, after correcting for age, sex, and education). For adjusted comparisons, we corrected the outcome variables using a linear regression with age, sex, and education as the predictors and then using the residuals. Two‐sample *t* tests are used for group comparisons. All *p* values are corrected using the Benjamini–Hochberg false discovery rate (FDR) correction. FDR‐corrected *q*  <  0.05 were considered statistically significant.

### 2.5 Standard protocol approvals, registrations, and patient consents

The study was approved by Committee for Medical and Health Research Ethics, Hospital Nacional Docente Madre‐Niño‐HONADOMANI “San Bartolomé” (12360‐18). Written informed consent was obtained from all patients and informants enrolled in the study and it was conducted in accordance with the Declaration of Helsinki.

## 3 RESULTS

The groups differed significantly in demographic and cognitive variables (Table 1). Age was highest in the AD group and lowest in the control group. Education levels were lowest in MCI and highest in FTD. Cognitive performance, measured by MMSE, RUDAS, and MoCA, was significantly lower in both AD and FTD compared to controls and MCI, with no significant difference between the two dementia groups.

**TABLE 1:** Demographic characteristics.

Demographic and clinical characteristics	Control	MCI	AD	FTD	Significance (*p* < 0.05)
*N* (%F)	781 (71%)	227 (66%)	315 (72%)	122 (58%)	FTD < MCI < Control = AD
Age	65.2 (8.0)	72.5 (8.4)	75.1 (8.3)	67.2 (8.7)	Control < FTD < MCI < AD
Education	11.0 (6.1)	8.1 (6.5)	11.1 (5.7)	12.7 (4.4)	MCI < Control = AD < FTD
MMSE	26.3 (3.8)	25 (5.2)	19.9 (4.9)	19.7 (6.4)	AD = FTD < Control = MCI
RUDAS	25.7 (2.6)	–	19.2 (5.2)	18.8 (7.2)	AD = FTD < Control
MoCA	26.3 (2.3)	–	12.2 (4.1)	13.9 (6.8)	AD < FTD < Control
Mild dementia	–	–	196 (62%)	75 (61%)	AD = FTD

*Note*: Data presented as mean (standard deviation) unless otherwise noted.

Abbreviations: AD, Alzheimer's disease; FTD, frontotemporal dementia; MCI, mild cognitive impairment; MMSE, Mini‐Mental State Examination; MoCA, Montreal Cognitive Assessment; RUDAS, Rowland Universal Dementia Assessment Scale.

**FIGURE 1:**
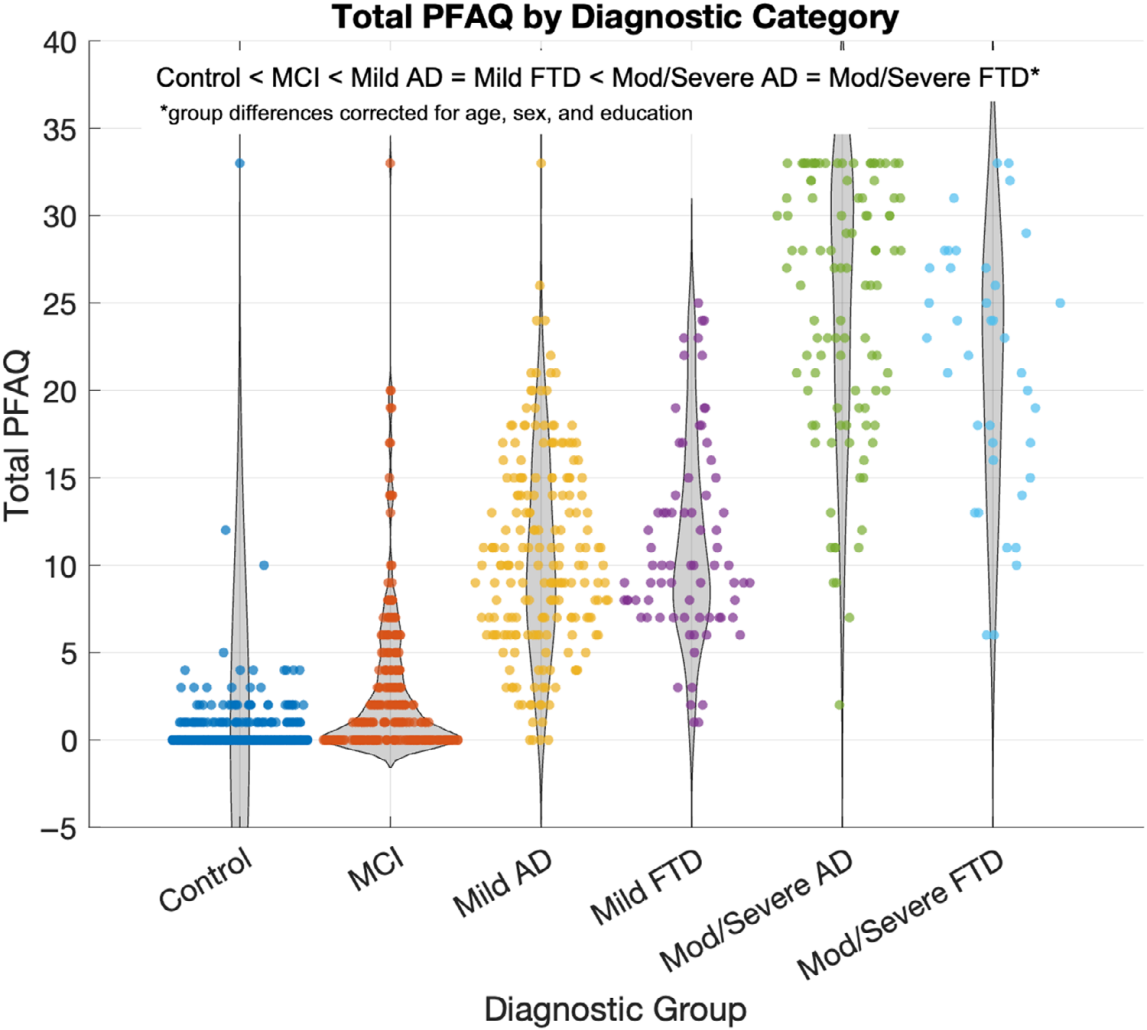
Total Pfeffer Functional Activities Questionnaire (PFAQ) score by diagnostic category. Violin plots with each colored dot represent an individual participant's score. AD, Alzheimer's disease; FTD, frontotemporal dementia; MCI, mild cognitive impairment; MOD, moderate.

Figure 1 illustrates the total PFAQ scores across diagnostic categories. Functional impairment increased progressively from cognitively normal individuals to those with moderate/severe dementia. MCI participants showed mild functional decline relative to controls, while all dementia groups (AD and FTD) showed markedly higher impairment (all FDR‐corrected *p* < 0.05). The highest PFAQ scores were observed in the moderate/severe AD and FTD groups. The Cohen's *d* for each group compared to controls were MCI: 1.03, mild AD: 3.6, mild FTD: 5.0, moderate/severe AD: 8.5, and moderate/severe FTD: 10.0.

**FIGURE 2:**
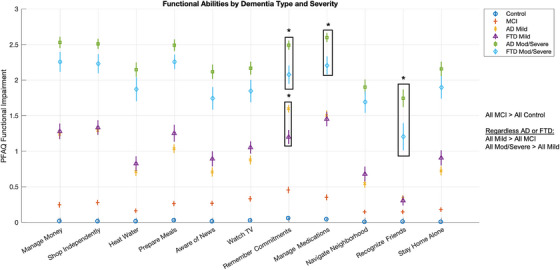
Item‐level analysis of functional abilities by dementia type and severity. Analysis for each question on the PFAQ. Each point represents the mean score for a specific functional activity, with error bars indicating variability. Asterisks denote statistically significant group differences for selected items. Boxed areas highlight items with the strongest discriminative value across severity groups. AD, Alzheimer's disease; FTD, frontotemporal dementia; MCI, mild cognitive impairment; MOD, moderate; PFAQ, Pfeffer Functional Activities Questionnaire.

Figure 2 presents comparisons of item‐level PFAQ scores across dementia types and severity levels. AD patients were most impaired in memory‐dependent activities, such as managing finances, remembering dates, and navigating unfamiliar places. In contrast, FTD patients showed disproportionate difficulties with executive and socially oriented tasks, including preparing meals, engaging in conversation, and performing complex planning. When stratifying by dementia severity, both AD and FTD showed marked increases in functional impairment from mild to moderate/severe stages. In AD, moderate/severe cases exhibited significantly higher total PFAQ scores than moderate/severe FTD cases, with notable worsening in memory‐related tasks, particularly in remembering commitments (Cohen's *d* = 0.59), managing medications (Cohen's *d* = 0.57), and recognizing friends (Cohen's *d* = 0.47), and the FDR‐corrected *p*‐value for these is <0.05; meanwhile, mild‐stage AD patients, compared with mild FTD, display impairment with remembering commitments (Cohen's *d* = 0.51, FDR‐corrected *p* < 0.05). In FTD, functional deterioration with severity showed a trend without significance to difficulties with executive and socially oriented tasks, including preparing meals, engaging in conversations (being aware of news and watching television [TV]), and staying home alone. Although both diseases showed progression in overall functional decline, the profile of item‐level impairments remained distinct by diagnosis, even within the same severity level, highlighting disease‐specific trajectories of functional loss.

**FIGURE 3:**
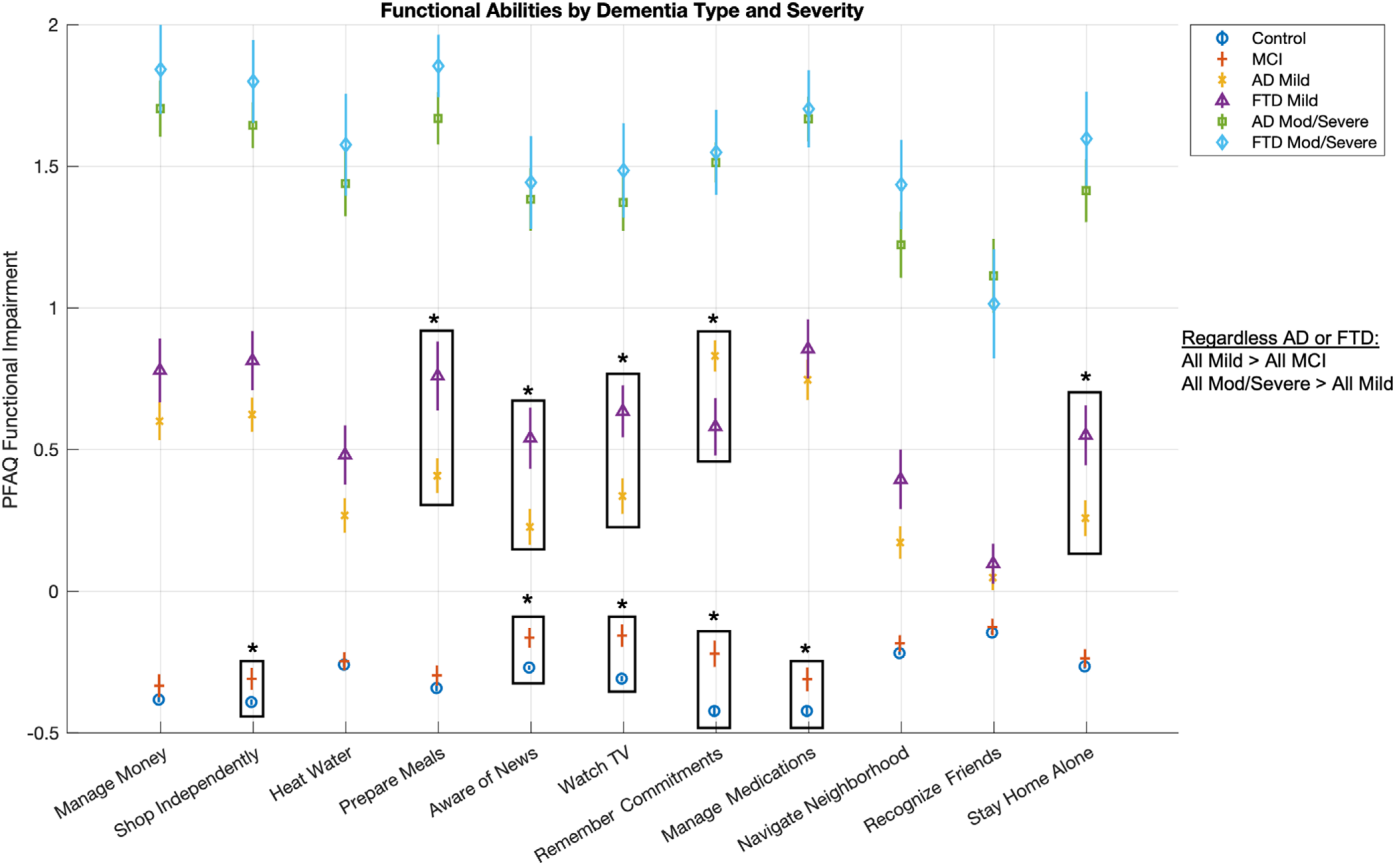
Item‐level analysis of functional abilities by groups and severity and adjusted for age, sex, and education. Each question is corrected for age, sex, and education using linear regression. Asterisks indicate statistically significant group differences (*p < 0.05), with boxed comparisons highlighting key discriminative items. AD, Alzheimer's disease; FTD, frontotemporal dementia; MCI, mild cognitive impairment; MOD, moderate; PFAQ, Pfeffer Functional Activities Questionnaire.

After adjusting for age, sex, and education, the corrected comparisons (Figure 3) retained the main findings. AD and FTD patients exhibited distinct patterns of functional impairment despite similar levels of global cognitive decline. In mild stages, AD continued to show greater deficits in memory‐linked items (e.g., impairment with remembering commitments; Cohen's *d* = 0.31, FDR‐corrected *p* < 0.05), while FTD patients demonstrated more executive and interpersonal functional impairments (preparing meals, being aware of news, watching TV, and staying home alone [Cohen's *d* > 0.33]). The difference between AD and FTD diminished at higher CDR levels (worse disease severity). Interestingly, some IADLs related to executive and socially oriented tasks could discriminate MCI from controls: shopping independently, being aware of news, watching TV, and others with memory‐related tasks: remembering commitments and managing medications. These differences remained statistically significant (Cohen's *d* > 0.21, FDR‐corrected *p* < 0.05) after sociodemographic adjustment. No significant interactions between sex or education and dementia subtype were found.

## 4 DISCUSSION

Our study set out to characterize functional capacity across the continuum from normal aging to MCI and two subtypes of dementia: AD and FTD in a Peruvian cohort, with a particular focus on distinguishing the profiles of AD and FTD. We found that functional activity impairments increased progressively with diagnostic severity. Even MCI and mild dementia cases had significant deficits in IADLs compared to controls. Notably, FTD patients demonstrated a distinct pattern of functional impairment relative to AD patients. While total PFAQ scores were elevated in both dementia groups, people with FTD tended to have greater impairment on complex domestic or community activities compared to people with AD. By documenting how and when daily abilities decline in AD versus FTD, our study provides clinically relevant insights that can improve differential diagnosis and care in an understudied population.^5^

Our results quantify what is expected: that AD^26^ and FTD^27^ display more functional deficits than controls and MCI, highlighting that the onset of dementia is accompanied by loss of independence in daily activities.^4^ Furthermore, we observed that FTD and AD had distinguishable functional profiles, which reflected their differing cognitive‐behavioral syndromes.^27^, ^28^ However, these differences diminished at more severe stages of dementia. Consistent with small^29^ and large longitudinal^30^ studies, FTD patients performed worse than AD in virtually all daily activities at baseline and showed a steeper longitudinal decline specifically in complex tasks like using appliances (e.g., a stove) and navigating travel. Our cross‐sectional findings mirror this pattern: Caregivers of FTD patients often reported more pronounced difficulty with multistep or planning‐intensive IADLs,^28^, ^29^ whereas AD patients showed profound deficits in memory‐dependent activities (such as remembering appointments or managing medications), in line with the hallmark memory impairment of AD.^26^, ^31^ Notably, we did not differentiate behavioral versus language variants of FTD, which may be an interesting future avenue to explore. We also found evidence of a hierarchical pattern of functional decline: Higher‐order IADLs (finances, medication management, meal preparation, transportation) were affected early and disproportionately, whereas more basic activities likely deteriorated later, consistent with the literature on staged loss of daily function.^32^, ^33^

Our findings also highlight the importance of caregiver‐informed functional assessments as part of dementia evaluations.^34^ Informant‐reported functional changes can sometimes even outpace measurable cognitive decline, indicating that tools like the PFAQ capture meaningful decline that cognitive tests might underestimate.^5^, ^34^ This is especially relevant given that functional impairment is a core criterion distinguishing dementia from prodromal stages. In addition, our results have implications for caregivers and care planning. Knowing the expected order of which tasks may require assistance helps caregivers prepare. Also, differentiating subtypes can be important, since the executive‐driven functional decline in FTD can impose a particularly high burden on caregivers, often exceeding the burden in AD.^35^, ^36^ Providing more knowledge sooner helps to empower support systems.

This study has several strengths. First, we analyzed a large, well‐characterized sample spanning the full cognitive spectrum, which provided robust power to detect even subtle group differences. This inclusivity (from healthy controls to MCI and mild and moderate/severe dementia) enabled a granular comparison across disease stages.^26^, ^37^ Second, we employed a well‐validated functional measure administered to knowledgeable informants, enhancing the ecological validity of our data. Third, all diagnoses were established through comprehensive clinical and neuropsychological evaluations following standardized criteria, which strengthens the validity of the group classifications (particularly the distinction between AD and FTD diagnoses).^38^ Finally, we accounted for key confounding variables (age, sex, and education).

We acknowledge several limitations and outline directions for future research. The cross‐sectional design of our study captures a single snapshot in time, which limits inferences about the rate of functional decline. Longitudinal studies are needed to confirm the trajectory of functional losses in AD^26^ and FTD.^28^, ^29^ Given that FTD is generally known to progress more rapidly than AD,^30^, ^39^ a prospective follow‐up would likely reveal even more pronounced divergence in how quickly everyday abilities deteriorate. Following patients over time could also establish whether specific early IADL deficits (such as those identified in our item analysis) predict subsequent cognitive decline or conversion from MCI to dementia.^40^, ^41^ Our reliance on a single informant‐reported questionnaire could introduce reporting biases. Caregivers might overestimate or underestimate a patient's difficulties due to their own stress or perspective, and future work should investigate inter‐rate variability. We did not differentiate between subtypes of FTD in this analysis. The category “FTD” largely consisted of behavioral variant FTD but may have included some language‐variant cases. Future studies with larger FTD samples should examine functional deficits by subtype to see if certain daily skills are preserved or lost differentially (e.g., patients with aphasic FTD might manage finances longer but struggle with communication‐related tasks).^39^ Our sample was drawn from a specialized memory clinic in an urban Peruvian setting, which may affect generalizability, particularly to rural or lower‐education populations. Most participants had a medium level of education and a certain degree of access to medical care at a private clinic, so it is possible that our findings cannot be directly extrapolated to more rural or lower‐income populations. Many of the control participants had subjective cognitive complaints (some simply wanted a cognitive evaluation to establish a normal baseline), so this may be a particular subgroup on the cognitive continuum that is not completely “normal,” which may explain the few control outliers. Also, these outliers may be due to non‐cognitive functional impairments, such as musculoskeletal disease, although we did our best to assess only impairments due to cognitive deficits. MCI is also a heterogeneous group, and future studies should differentiate amnestic MCI from non‐amnestic MCI. Within our dementia groups, CDR is a relatively coarse rating system, and a more continuous cognitive scale might help to elucidate subtle differences on the continuum of cognitive decline. Cultural factors might also influence functional assessments, thereby affecting informants’ ratings. It will be important for future research to replicate these findings in community‐based cohorts and in other Latin American populations to ensure broad applicability. Finally, although we adjusted for demographics, other potential confounders (comorbid medical illnesses, depression, or variability in caregiver characteristics) were not analyzed and could contribute to functional impairment. Future work might incorporate these factors, as well as biomarkers, to explore clinical–biological correlations.

## 5 CONCLUSIONS

This study underscores the idea that functional decline is a core feature of dementia that manifests in syndrome‐specific ways in Peruvian populations. Patients with AD and FTD experience different cognitive symptoms but also differ in how their daily lives are impacted, a distinction that can be detected with caregiver‐based functional measures. By comparing AD and FTD in a Peruvian cohort, we have shown that assessing what daily tasks are impaired, and how they are impaired, provides valuable information beyond cognitive testing alone. These insights can inform earlier and more accurate diagnoses, as well as personalized strategies to preserve independence. Ultimately, our findings emphasize that maintaining instrumental functional abilities is critical for patient quality of life and caregiver well‐being, reinforcing the need to make functional assessment and support an integral part of dementia care and research.

## AUTHOR CONTRIBUTIONS

Nilton Custodio: Conceptualization; methodology; writing; critical revision; final approval. Gregory Brown: Conceptualization; methodology; analysis; critical revision; final approval. Belen Custodio: Methodology; writing; critical revision; final approval. Rosa Montesinos: Conceptualization; methodology; critical revision; final approval. Milagros Huanca: Conceptualization; critical revision; final approval. Pamela Bartolo: Conceptualization; critical revision; final approval. Zadith Yauri: Conceptualization; critical revision; final approval. Katherine Agüero: Conceptualization; critical revision; final approval. Graciet Verastegui: Conceptualization; critical revision; final approval. Agustin Ibañez: Conceptualization; methodology; analysis; writing; critical revision; final approval.

## CONFLICT OF INTEREST STATEMENT

The authors declare no conflict, of interest. Author disclosures are available in the Supporting Information.

## CONSENT STATEMENT

All participants participated voluntarily in the study and provided written informed consent.

## Supporting information



Supporting Information
